# Towards a comprehensive biomechanical assessment of the elderly combining *in vivo* data and *in silico* methods

**DOI:** 10.3389/fbioe.2024.1356417

**Published:** 2024-05-06

**Authors:** Giorgio Davico, Luciana Labanca, Irene Gennarelli, Maria Grazia Benedetti, Marco Viceconti

**Affiliations:** ^1^ Department of Industrial Engineering, Alma Mater Studiorum - University of Bologna, Bologna, Italy; ^2^ Physical Medicine and Rehabilitation Unit, IRCCS Istituto Ortopedico Rizzoli, Bologna, Italy; ^3^ Department of Electronics and Telecommunications, Politecnico di Torino, Torino, Italy; ^4^ Department of Biomedical and Neuromotor Sciences, Alma Mater Studiorum - University of Bologna, Bologna, Italy; ^5^ Medical Technology Lab, IRCCS Istituto Ortopedico Rizzoli, Bologna, Italy

**Keywords:** biomechanical assessment, MVIC, dynamometry, SNMES, musculoskeletal modeling, aging population

## Abstract

The aging process is commonly accompanied by a general or specific loss of muscle mass, force and/or function that inevitably impact on a person’s quality of life. To date, various clinical tests and assessments are routinely performed to evaluate the biomechanical status of an individual, to support and inform the clinical management and decision-making process (e.g., to design a tailored rehabilitation program). However, these assessments (e.g., gait analysis or strength measures on a dynamometer) are typically conducted independently from one another or at different time points, providing clinicians with valuable yet fragmented information. We hereby describe a comprehensive protocol that combines both *in vivo* measurements (maximal voluntary isometric contraction test, superimposed neuromuscular electrical stimulation, electromyography, gait analysis, magnetic resonance imaging, and clinical measures) and *in silico* methods (musculoskeletal modeling and simulations) to enable the full characterization of an individual from the biomechanical standpoint. The protocol, which requires approximately 4 h and 30 min to be completed in all its parts, was tested on twenty healthy young participants and five elderlies, as a proof of concept. The implemented data processing and elaboration procedures allowing for the extraction of several biomechanical parameters (including muscle volumes and cross-sectional areas, muscle activation and co-contraction levels) are thoroughly described to enable replication. The main parameters extracted are reported as mean and standard deviation across the two populations, to highlight the potential of the proposed approach and show some preliminary findings (which were in agreement with previous literature).

## 1 Introduction

As we age, our muscles tend to lose mass and to reduce in volume/size (Clark and Manini, 2008; 2010; Seene and Kaasik, 2012; Clark, 2019). This natural process, referred to with the term sarcopenia, may be accelerated or aggravated by ongoing neural diseases or musculoskeletal disorders (Fukagawa et al., 1995; Kemmler et al., 2015). For decades, sarcopenia has been identified as the major determinant of physical frailty in the elderly, and often used as univocal term to define the typical loss of muscle strength observed in the aging population. In the early 2000s, the paradigm changed, as it became apparent that muscles did not simply (or necessary) exhibit a reduced strength secondary to a loss of mass. Thus, a new term was introduced in the attempt to describe the complexity of the phenomenon of the loss of muscle strength: dynapenia. Today, with the term dynapenia, clinicians and research scientists refer to all those factors associated with the loss of muscle strength other than sarcopenia, e.g., changes in contractile properties or neural activation impairments (Clark and Manini, 2008). Although the decline in muscle function in the elderly is commonly related to a combination of sarcopenia and dynapenia, several studies have shown that the two can manifest independently on one another and that usually the decline in muscle strength is more rapid than the concomitant loss of muscle mass (Frontera et al., 2000; Delmonico et al., 2009). Furthermore, a number of pathological conditions typical of the elderly, such as osteoarthritis, may lead to further impairments in the neural system which go beyond the neural alterations common to the aging process (Rice et al., 2011). Thus, it is clear that the conundrum is not only on the semantics. The distinction between sarcopenia and dynapenia is necessary to appropriately identify and quantify the causes and impairments related to muscle weakness, and in turn to improve the clinical management of the elderly. In fact, several rehabilitation/nutritional/pharmacological interventions may be better set up to address either the loss of muscle mass or the abnormalities in neural function. To date, the clinical assessment is based on a number of measures which comprehensively assess muscle weakness, thus leaving uncertainty on muscle weakness determinants which are majorly affected by the aging process (Dent et al., 2018; Beaudart et al., 2019).

The gold standard measures to quantify the (loss of) muscle volume and the residual muscle force of a person are—respectively—the acquisition of medical imaging data (typically magnetic resonance images, MRI) to extract muscle volumes (Barnouin et al., 2014; Pons et al., 2018; Davico et al., 2023) and the maximal voluntary isometric contraction (MVIC) test on a dynamometer (Meldrum et al., 2007; O’Brien et al., 2009; Harbo et al., 2012). Alternatively, the outcome of a hand-grip test may be employed as a surrogate measure of generalized sarcopenia (Porto et al., 2019; Lee and Gong, 2020), and bio-impedance devices may be used to estimate the body mass composition (Khalil et al., 2014). Last, the activation inhibition may be identified with a superimposed electrical stimulation (Clark and Taylor, 2011). While informative, these measures—each requiring a specific instrumentation or not common in the clinical practice, thus often performed separately—do not provide a full characterization of the biomechanical determinants of dynapenia. Nonetheless, leveraging on these common clinical examinations, and complementing the assessment with novel musculoskeletal (MSK) modeling techniques it is possible to achieve a complete characterization of the health status of an individual from a biomechanical standpoint.

Therefore, we set out to design an experimental and computational protocol which provides information on various key domains, from the loss of muscle mass (sarcopenia) and force (dynapenia), to inhibited or altered muscle activation and internal biomechanics. Our aim was threefold: 1) to develop a comprehensive framework which combines *in vivo* assessments and *in silico* simulations to fully characterize the biomechanics of an individual, 2) to test the feasibility of the developed framework in a clinical context and on an elderly population and 3) to highlight any correlations between the various measured or predicted biomechanical parameters.

## 2 Methods and protocol

The data and results hereby presented have been developed in the framework of the Proto-Aging project. The study protocol was approved by the local Ethical Committee (CE AVEC: EM37/2023_30/2021/Sper/IOR_EM2) and has been recorded on the ClinicalTrials registry (ClinicalTrials ID: NCT05854316). The study was conducted in accordance with the Declaration of Helsinki and a written informed consent was signed by each of the participants before participating in the study.

### 2.1 Participants

Twenty healthy young individuals (age: 28.39 ± 4.97 years, BMI: 22.19 ± 2.79 kg/m2) and five elderlies (age: 68.04 ± 2.01 years, BMI: 25.57 ± 2.73 kg/m2) participated in the study (Table 1). Were excluded from the study subjects who had 1) neurological, rheumatic or tumoral diseases, 2) present or previous injuries to bones, muscles and tendons of the lower limbs which required surgical interventions or led to abnormalities in the structure or the function of the lower limb, 3) sedentary behavior as defined by level 1 of the Saltin and Grimby physical activity level scale (Grimby et al., 2015), and/or 4) physical conditions or health issues not compliant with MRI assessment and electrical stimulation use.

**TABLE 1:** Participants demographics, including height, mass, body mass index (BMI), age, sex and physical activity (PA) level. The PA level is a 1 to 4 score according to the Saltin-Grimby scale (Grimby et al., 2015), where 1 = sedentary; 2 = light physical activity; 3 = moderate activity; 4 = agonistic level. Pre = previous PA level, cur = PA level at the time of the visit.

ID	Height (m)	Mass (kg)	BMI (kg/m^2^)	Age (years)	Sex	PA level	
						pre	cur
**HYA01**	1.70	83.00	28.72	26.64	M	3	3
**HYA02**	1.60	54.00	21.09	27.41	F	3	3
**HYA03**	1.73	73.00	24.39	40.95	M	3	2
**HYA04**	1.70	59.00	20.42	27.93	M	4	3
**HYA05**	1.77	68.00	21.71	27.12	M	4	4
**HYA06**	1.84	70.00	20.68	23.27	M	4	3
**HYA07**	1.75	75.00	24.49	29.52	M	3	3
**HYA08**	1.66	63.00	22.86	38.92	F	4	4
**HYA09**	1.65	45.00	16.53	23.26	F	3	3
**HYA10**	1.77	77.00	24.58	33.57	M	4	3
**HYA11**	1.54	50.00	21.08	34.29	F	3	3
**HYA12**	1.63	50.00	18.82	23.07	F	3	3
**HYA13**	1.81	83.00	25.34	26.67	M	4	4
**HYA14**	1.70	55.00	19.03	27.83	F	3	2
**HYA15**	1.78	75.00	23.67	23.85	M	4	4
**HYA16**	1.69	59.00	20.66	30.82	F	4	3
**HYA17**	1.92	82.00	22.24	22.21	M	4	4
**HYA18**	1.81	68.00	20.76	26.95	F	4	3
**HYA19**	1.58	51.00	20.43	27.14	F	4	4
**HYA20**	1.55	63.00	26.22	26.46	F	3	3
**OLD01**	1.73	80.00	26.73	70.13	M	3	2
**OLD02**	1.80	78.00	24.07	64.96	M	3	3
**OLD03**	1.80	84.00	25.93	69.69	M	3	2
**OLD04**	1.70	62.00	21.45	66.40	F	3	3
**OLD05**	1.58	74.00	29.64	69.03	F	3	3

### 2.2 Study protocol

**FIGURE 1:**
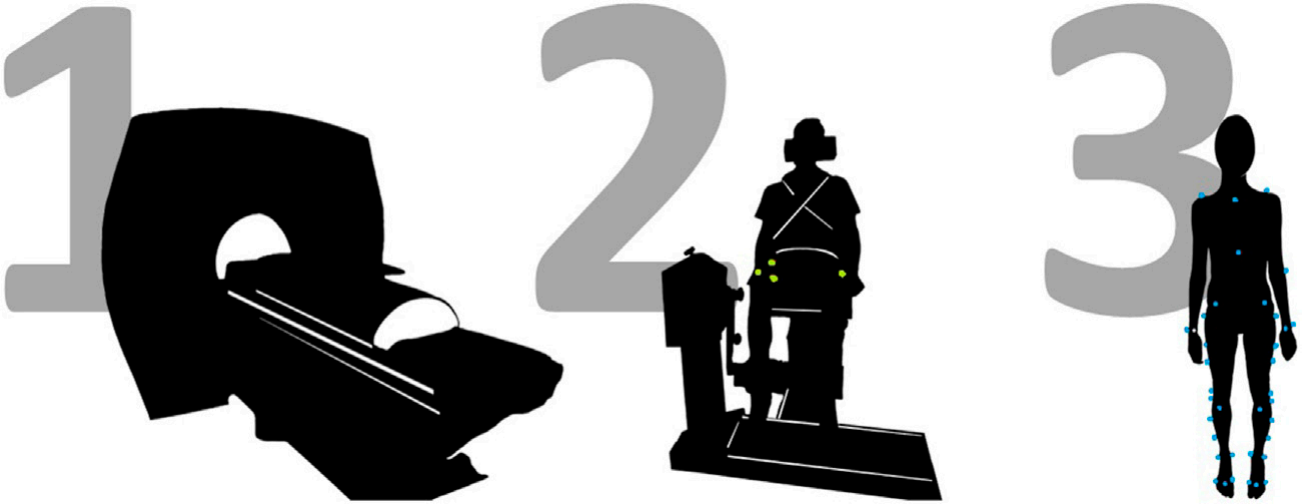
Schematic representation of the study protocol, involving three different data acquisition sessions. First, full lower limb MRI data are acquired on a 3T scanner. Then, a MVIC test is performed on a dynamometer, in various knee configurations (i.e., with knee flexed at 75° and 90° degrees), whilst recording surface EMG data from eight major lower limb muscles. This includes the delivery of a superimposed neuromuscular electrical stimulation. Last, a gait assessment in conducted.

The experimental data collection was organized in three separate sessions, to respectively collect full lower limb MRI data, maximal voluntary and involuntary knee extension and flexion torques and to perform a full gait assessment (Figure 1).

#### 2.2.1 MRI data acquisition

With the subjects in supine position, full lower limb MRI data, from the 4th lumbar vertebra (L4) to the toes, were acquired on a 3T MRI scanner (DISCOVERY MR750w with XP, GE Healthcare, Chicago, IL-USA), using a Dixon sequence specifically optimised to highlight muscle boundaries (slice thickness: 2 mm, Minimum overlap: 20 slices, matrix size: 240 × 240 px, TR: 3.74 ms, TE: 2.2 ms, NEX: 1). Axial images were acquired in multiple separate stations (4 stations for people below 170 cm, 5 stations for people taller than 170 cm). A footrest was used to ensure that the ankles were in neutral position, i.e., with the angle between foot and tibial bone approximately at 90°.

#### 2.2.2 Muscle force assessment

On a separate day, the subjects were asked to perform a maximal voluntary isometric contraction (MVIC) test to quantify the muscle force of the knee extensor and flexor muscles.

##### 2.2.2.1 Subject preparation

At subject’s arrival, the operators identified the placement sites for surface EMG electrodes on eight primary lower limb muscles (i.e., rectus femoris - RF, vastus medialis—VM, vastus lateralis—VL, caput longum of the biceps femoris—BFL, semitendinosus—ST, lateral gastrocnemius LG. EMG data from the VL and BFL were collected on both sides), according to SENIAM recommendations (Hermens et al., 2000). Following skin preparation (shaving and cleaning), pairs of Ag-AgCl EMG electrodes (10 mm diameter) were placed on the selected locations with a 20 mm interelectrode distance (centre-to-centre).

##### 2.2.2.2 Muscle strength assessment

Prior to skin preparation, the subjects were asked to sit on the physiotherapy bed and to perform a hand-grip test. Holding a hydraulic dynamometer (Jamar) in their dominant hand, with the elbow flexed at 90°, the participants performed three maximal contractions. After each contraction, the maximum force value, in kg, was noted and a 90 s break was allowed for the arm to rest. Verbal encouragement was provided to elicit maximal contractions. If the level of force kept increasing, a fourth attempt was requested.

Upon electrodes placement, and prior to the actual MVIC test, subjects performed a 10-min warm-up session on an ergometer at low resistance. Then, one electrogoniometer (Biometrics Ltd., Gwent, United Kingdom) was placed on the lateral side of the dominant limb with the two arms aligning with the thigh and leg axes. Both the electrogoniometer and the bipolar electrodes were connected through cables to the EMG system (Sessantaquattro, OTBioelettronica, Torino, Italy. Sampling frequency: 2000 Hz). Baseline EMG data were collected with the subjects sitting on a chair and relaxing for 30 s.

Subsequently, the subjects were seated on an isokinetic dynamometer (Biodex System 4 Pro, Biodex Medical Systems, New York, United States) and strapped to the chair using belts across the chest, waist, thigh and ankle of the dominant (assessed) limb. With the dynamometer arm at 75° flexion, the subjects were asked to perform a series (*n* = 8) of short (i.e., 3 s) sub-maximal contractions at 50% of self-perceived maximal effort. Consecutive contractions were separated by a 2-s pause.

Following warm-up, the subjects were asked to perform a MVIC test in different configurations (i.e., with the knee flexed at 75° and 90°, where 0° is full extension), first in extension then in flexion. To execute the test, all participants were instructed to exert their maximal strength as fast as possible. Verbal encouragement (e.g., Go!, Forza!) and visual feedback were provided to elicit maximal contractions and to help participants maintaining the level of force for at least 3 s before relaxing. Three trials were performed at each angle and for each muscle group (knee extensors and knee flexors). If the force expressed (i.e., the measured torques) in the third trial exceeded by 5% or more the values achieved in the previous trials, the participants were asked to perform a fourth trial (Ditroilo et al., 2010; Senefeld et al., 2017). To avoid muscle fatigue, a 90 s resting period was allowed between contractions.

##### 2.2.2.3 Neuromuscular electrical stimulation

Quadriceps muscle voluntary activation deficit was assessed by means of superimposed neuromuscular electrical stimulation (SNMES). While the participants performed a maximal contraction, with the knee flexed at 75°, a doublet of single square-wave stimuli (2 square pulses; interpulse interval: 10 ms; pulse duration: 100-µs; maximal voltage: 330 V; intensity: from 200 to 500 mA) were delivered by a constant current high-voltage stimulator (Digitimer DS7AH, Hertfordshire, United Kingdom), through a couple of reusable synthetic chamois leather electrodes (FIAB spa, Vicchio, Italy) applied over the thigh. The size of the electrodes (12 × 8 or 21 × 11 cm) was chosen according to each participant’s size to warrantee the stimulation of a representative portion of the quadriceps muscle. The intensity of the stimulation was determined in accordance with previous studies (Malloggi et al., 2019; Catino et al., 2021). First, the peak torque was recorded during MVIC contraction with the knee flexed at 75°; then, the subjects were asked to rest and relax their muscles. To identify an adequate intensity for each participant, stimulations at increasing intensity (of current), and with a 2-min rest between, were delivered until a torque value equal to or greater than 25% of the recorded peak torque (MVC25) was observed. Once the intensity of stimulation had been selected, the participants were asked to perform three MVIC of the knee extensor muscles. The stimulation was delivered when the plateau (maximum torque level) was reached and maintained for at least 2 s. A resting period of 2 min between repetitions was granted to avoid muscle fatigue. The dynamometry and SNMES data were synchronized through the Power1401 data acquisition system (CED, Cambridge, United Kingdom), and visualized and recorded in Spike II v10 (Cambridge Electronic Designed Limited-CED, Cambridge, United Kingdom).

#### 2.2.3 Gait assessment

On the same day or later, the participants underwent a full gait assessment.

An expert operator removed eventual hair from the skin which was then cleansed with ethyl alcohol to reduce impedance. Then the correct location for the Ag-AgCl EMG electrodes was identified in accordance with SENIAM recommendations. Overall, 16 muscles were identified (9 on the limb of interest—i.e., soleus, tibialis anterior, gluteus medius, and the six acquired during the MVIC test—and 7 on the contralateral side. The EMG signals from the soleus and gluteus medius muscles were solely collected on the leg of interest) and wireless EMG sensors (EMG Wave, Cometa^®^, Milan, Italy) were placed and secured in position with a hypoallergenic double-sided tape. Then, 49 retro-reflective spherical markers were placed on anatomical landmarks on the upper body (torso, spine, shoulders, arms) and lower limbs (pelvis, femurs, tibiofibular complexes and feet) (Leardini et al., 2007), as well as on thighs and shanks.

Once instrumented, the subjects were asked to perform few simple locomotor tasks, including 1) one calibration trial with the subjects standing in T-pose in front of the cameras (16 camera system, Vicon), and 2) a minimum of 10 walking trials over a 10 m walkway at self-selected (habitual) walking speed. The trials were deemed acceptable if characterized by clean strikes on the force plates (Kistler, Kistler Instrumente AG, Winterthur, Switzerland).

## 3 Data analysis

### 3.1 Muscle volumes

The axial MRI scans, saved in DICOM format, were imported in the Mimics Innovation Suite software (Materialise, Leuven, BE), and organized to ensure consecutive stacks of images were merged together. Then, using the software Mimics (v23, Mimics Innovation Suite, Materialise, Leuven, Belgium), the volumes of the major knee extensor and flexor muscles of the limb of interest, respectively the quadriceps (vastus medialis, vastus lateralis, vastus intermedium, and rectus femoris) and the hamstrings (semitendinosus and biceps femoris) were semi-automatically segmented, and exported in STL file format. A custom written python script was ultimately run to extract all muscle volumes and maximal cross-sectional areas (CSA).

### 3.2 Maximal isometric torques

The experimental data collected during the MVIC test, originally saved in a proprietary format (OTB), were exported in MATLAB files from within the OTBiolab+ software v1.5.7 (OT Bioelettronica, Turin, Italy). In MATLAB 2022b, the torques data were first filtered with a zero-phase shift 4th order Butterworth lowpass filter (fcutoff = 20 Hz) and converted in Nm:

$$T_{\text{Nm}}={Out}_{dyn}\times f_{V\_\text{Ft}-\text{lb}}\times f_{\text{Ft}-\text{lb}\_\text{Nm}}={Out}_{dyn}\left[V\right]\times 102.4\;\left[\frac{ft-lb}{V}\right]\times 1.3558\;\left[\frac{Nm}{ft-lb}\right]={Out}_{dyn}\times 138.834$$

where Out_dyn_ is the output torque from the dynamometer (in V), while f_V_Ft-lb_ and f_Ft-lb_Nm_ are the conversion factors from Volts to foot-pounds (according to the vendor) and from foot-pounds to Nm, respectively.

Then, for each subject, task and knee configuration, the dynamometry data were segmented to separate the different trials (repetitions). To this end, a threshold value was set to identify the start and end of the contraction (i.e., the analysis window), as follows:

$${threshold=\mu }_{noise}+3\sigma_{\text{noise}}$$

where μ_noise_ and σ_noise_ are, respectively, the mean and standard deviation of the signal where, according to the protocol, there was no contraction (i.e., noise).

Last, a zero-phase shift 4th order Butterworth lowpass filter at 2 Hz was applied to smooth the signal, so to avoid overestimation of the maximal torques due to sudden bursts, and the maximum value was extracted. The overall maximum among the three trials (per task and configuration) was considered to be the MVIC torque value.

### 3.3 Central activation ratio

A similar approach was employed to quantify any activation deficit, on the dynamometry data recorded while the SNMES was delivered. The dynamometry data were filtered with a low-pass Butterworth filter at 20 Hz, and the offset removed. The Central Activation Ratio (CAR) was calculated as in (Kent-Braun and Le Blanc, 1996):

$$CAR=\left(\frac{MVC}{MVC+superimposed\;stimulus}\right)\times 100$$

where MVC is identified as the torque value occurring in correspondence of the plateau, right before the stimulation, while the stimulated torque is derived from the amplitude of the superimposed twitch.

For this analysis, only the trials where the electrical stimulation was delivered during the maximum voluntary contraction were considered.

### 3.4 Muscle activity

The EMG signals recorded while the subjects performed the MVIC test and the dynamic tasks (e.g., overground walk) during the warm-up phase prior to the test, were filtered within the 20–300 Hz band using a zero-lag 5th order Chebyshev high-pass filter followed by a 8th order Chebyshev lowpass filter (Merletti and Cerone, 2020). A recursive filter was then applied to remove the 50 Hz noise frequency and its higher harmonics. The filtered signal was thus rectified and lowpass filtered with a 2 Hz zero-lag 4th order Butterworth filter to extract its linear envelope, which was later normalized by the maximum value observed during the task. Finally, the co-contraction index (CCI) was computed as suggested by Rudolph and colleagues (Rudolph et al., 2000):

$$CCI\left(t\right)=\left(\frac{{input}_{L}}{{input}_{H}}\right)\times \left({input}_{L}+{input}_{H}\right)$$

where input_L_ and input_H_ are respectively the activation levels of the less active and more active antagonist muscle (between BFL and VL) during the execution of the analyzed task.

For the overground walking trials, the CCI was computed separately for each of the four phases of the gait cycle (i.e., initial double support—0%–10%, single support: 10%–50%, pre-swing: 50%–60%, swing phase: 60%–100%). Of note, the CCI ranges from 0 to 2.

### 3.5 Musculoskeletal modeling and simulations

All data collected during the gait assessment were pre-processed in Vicon Nexus, where any gaps due to partial marker occlusion were filled to ensure continuous marker trajectories. Then, the data were exported in c3d file format and later processed in MOtoNMS (Mantoan et al., 2015), to obtain one file containing the data from the force plates and one file containing the trajectories of all markers (respectively, in MOT and TRC file format), ready for use in the OpenSim software (Delp et al., 2007; Seth et al., 2018).

For each subject, a musculoskeletal model was generated by linearly scaling the generic modified full body model (Rajagopal et al., 2016; Uhlrich et al., 2022). Motion capture data from a single calibration trial where the subjects were standing in T-pose were used to guide the scaling process (i.e., to identify the scaling factors).

The models were employed within the OpenSim (v4.1) environment to perform biomechanical simulations of gait. For each participant, data from 10 overground walking trials were analyzed. Joint angles, external moments and contact forces were predicted via the Inverse Kinematics, Inverse Dynamics and Joint Reaction Analysis tools. Muscle forces and activations were estimated hypothesizing optimal muscle control, i.e., implementing a cost function to identify the solution that minimized the overall metabolic cost expenditure (sum of squared muscle activations) (Crowninshield and Brand, 1981).

All parameters (muscle volumes and CSAs, MVIC torque values in extension and flexion with the knee flexed at 75° and 90°, CAR and CCI during maximal contractions, hand-grip force, average walking speed, and hip, knee and ankle ROMs on the sagittal plane), extracted at participant’s level, were pooled together to compute the mean and standard deviation for the two populations under study (young and elderly individuals) (Table 3).

The OpenSim output were 1) normalized to each subject’s mass (joint moments) or body weight (joint contact forces), 2) interpolated to vectors of 101 points (0–100) to express the data in percentage of the gait cycle, and 3) organized in structures to facilitate subsequent analyses. Statistical Parametrical Mapping techniques (SPM, spm1d in Matlab)(Pataky, 2012) were employed to identify any statistically significant differences between the ankle, knee and hip joint kinematics, kinetics and contact force profiles of the two cohorts under study (i.e., healthy young individuals and elderlies). Significance for the t-tests was set to 0.05.

## 4 Results

All participants successfully completed the entire protocol. The average time to complete the MRI acquisition was 30 min, while approximately 1 h and 2 h were respectively allocated for the gait assessment and the MVIC test (mostly spent for the participants’ preparation and warm-up). Approximately 20 min were required to fully process the dynamometry data (i.e., sEMG and torques) for one subject, using custom written MATLAB scripts. Similarly, the elaboration of the gait data for each participant, which was fully automated through MOtoNMS (Mantoan et al., 2015), took on average 15 min. The segmentation of medical imaging data was performed with a semi-automatic (atlas-based) approach in Mimics v23 and took up to 6 h per subject to completely segment the muscles of one lower limb.

Due the breadth of the dataset and the main aim of this work, to keep this section brief and easy to read, only a subset of the data and results will be presented in the following. Our intent is to show the type of data and parameters that can be extracted following the proposed framework (Table 2, Table 3). For more comprehensive set of results, the reader is referred to the Supplementary Material and Data availability sections.

**TABLE 2:** Summary of the methods employed to process the experimental data and list of parameters thus extracted. BP = band-pass filter, CAR = central activation ratio, CCI = co-contraction index, EMG = electromyography, LP = low-pass filter, MRI = magnetic resonance image, MSK = musculoskeletal, MVIC = maximum voluntary isometric contraction, RMS = root mean squared, SNMES = superimposed neuromuscular electrical stimulation.

Data type	Elaboration method	Parameter extracted
**Surface EMG**	RMS envelope (BP 20–300 Hz, 50 Hz noise removal, rectification, LP 2 Hz)	CCI
**Torques**	Filtering (LP 20 Hz), segmentation (on/off signal to define window) Filtering (LP 2 Hz)	MVIC (overall max)
**MRIs**	Semi-automatic segmentation	Volumes (both sides)
**SNMES**	Filtering (LP 20 Hz), identification voluntary and involuntary torques	CAR
**MSK modeling**	Scaled generic models and biomechanical simulations in OpenSim	Joint kinematics, kinetics and contact forces

**TABLE 3:** Example of parameters that can be extracted from the experimental tests and *in silico* simulations. CAR = central activation ratio, JCF = knee joint contact force characteristic (1^st^/2^nd^) peak value, MVIC = maximal voluntary isometric contraction in extension, PWS = preferred walking speed, ROM = range of motion on the sagittal plane, Volume = quadriceps muscles volume, SS = single leg support phase.

Parameter	Unit	HYA	OLD
**MVIC**_**75°**_	**Nm**	236.1 ± 68.6	183.1 ± 44.4
**MVIC**_**90°**_	**Nm**	222.8 ± 76.1	160.6 ± 39.7
**CAR**	**-**	0.96 ± 0.04	0.98 ± 0.01
${\text{CCI}}_{75{}^{\circ}}^{\text{MVIC}}$	**-**	0.15 ± 0.21	0.13 ± 0.10
${\text{CCI}}_{\text{SS}}^{\text{Walk}}$	**-**	0.13 ± 0.07	0.31 ± 0.06
**Hand-grip**	**kg**	40.55 ± 8.93	36.80 ± 10.05
**Volume**	**cm** ^**3**^	1673.7 ± 475.1	1482.2 ± 127.0
**PWS**	**m/s**	1.25 ± 0.12	1.32 ± 0.09
${\text{ROM}}_{\text{Ankle}}^{\text{Sag}}$	**deg**	31.44 ± 6.14	28.88 ± 3.72
${\text{ROM}}_{\text{Knee}}^{\text{Sag}}$	**deg**	64.07 ± 4.13	58.85 ± 1.99
${\text{ROM}}_{\text{Hip}}^{\text{Sag}}$	**deg**	40.69 ± 4.34	37.55 ± 3.30
${\text{JCF}}_{\text{Knee}}^{1st\;\text{peak}}$	BW	3.08 ± 0.52	3.73 ± 0.57
${\text{JCF}}_{\text{Knee}}^{2nd\;\text{peak}}$	BW	3.14 ± 0.24	2.62 ± 0.30

### 4.1 Muscle volumes

The volumes of the quadriceps muscles, with the only exception of the VM, were smaller in the elderly participants compared to the young adults (e.g., 597.0 ± 162.3 cm^3^ vs. 510.1 ± 92.622 cm^3^, for the VL. Table 4). Similarly, in the HYA cohort, the maximal CSA of the RF, VL and VI muscles were larger than on the elderlies. Similar findings were observed when the volumes were normalized to the BMI of each participant, to remove the confounding effect of the height and mass.

**TABLE 4:** Muscle volumes reconstructed from the MRI data, expressed as mean and standard deviation across each cohort, and reported in cm^3^. RF = rectus femoris, VI/VL/VM = vastus intermedius/lateralis/medialis.

Muscle	Volume (cm^3^)		CSA_max_ (mm^2^)	
	HYA	OLD	HYA	OLD
**RF**	224.0 ± 75.4	117.8 ± 36.1	1233.5 ± 340.3	949.9 ± 189.1
**VL**	597.0 ± 162.3	510.1 ± 92.6	2807.4 ± 634.5	2421.1 ± 384.4
**VI**	430.2 ± 110.2	365.7 ± 87.9	2080.0 ± 496.6	1807.2 ± 461.4
**VM**	422.4 ± 127.2	432.5 ± 110.4	2283.3 ± 541.2	2284.2 ± 359.0

### 4.2 MVIC test

The MVIC test was successfully conducted on all participants, in both configurations (i.e., with the knee flexed at 75° and 90°). In general, with the knee flexed at 75° rather than 90°, the participants were able to generate their overall maximum extension torques (i.e., 236.1 ± 68.6 Nm and 183.1 ± 44.4 Nm, respectively, for the HYA and OLD cohort) (Table 5). The torques generated by the elderlies were the smallest overall.

**TABLE 5:** Maximum extension torques values (in Nm) and knee flexion angle (in degrees) measured by the dynamometer and the electrogoniometer (internal angle), respectively, for the cohort of young healthy adults (HYA) and elderly individuals (OLD), in the two tested configurations (with the dynamometer angle set at 75° and 90°). The values are reported as mean and standard deviation across the respective cohort.

	Maximal torques (Nm)		Internal angle (°)	
	HYA	OLD	HYA	OLD
**75°**	236.1 ± 68.6	183.1 ± 44.4	60.9 ± 8.6	57.3 ± 4.2
**90°**	222.8 ± 76.1	160.6 ± 39.7	72.8 ± 9.3	68.1 ± 7.1

### 4.3 Hand-grip

**FIGURE 2:**
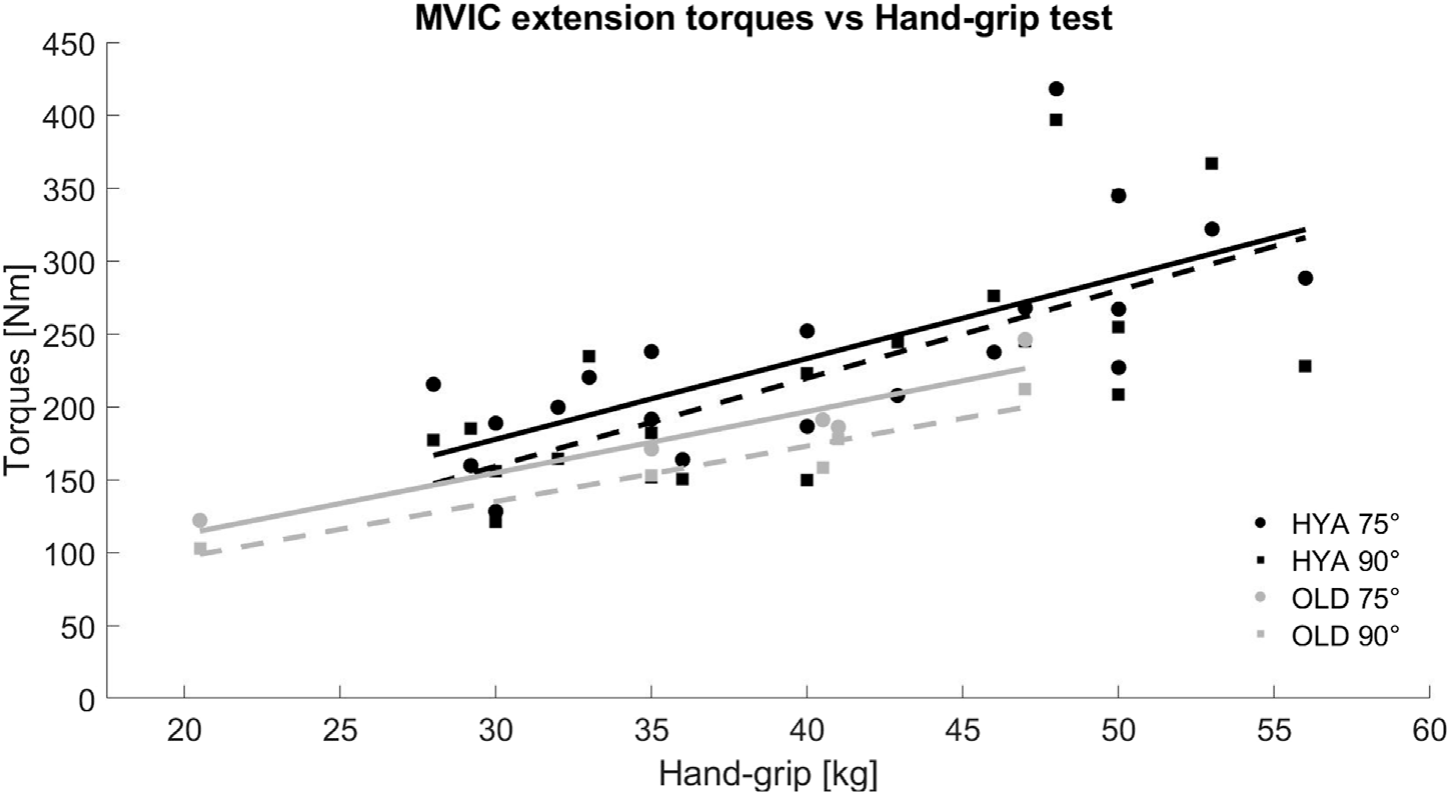
Relationship between the maximal extension torques recorded during the MVIC test in extension and the hand-grip test, for both young (HYA, black) and elderly (OLD, grey) participants. The solid and dashed lines represent the trend line of the data considering the results of the MVIC test performed with the knee flexed at 75° and 90°, respectively.

The results of the hand-grip test were in line with the MVIC test (i.e., HYA_males_>HYA_females_>OLD), with values ranging between 40 and 56 kg for young males, between 28 and 40 kg for young females, and between 20.5 and 47 kg for the elderlies. A positive and linear relationship was found between the maximal extension torques and the maximal strength measured with the hand-grip test (Figure 2).

### 4.4 Muscle activity and activation deficit

All participants were able to fully recruit their muscles voluntarily, as highlighted by the CAR values (larger than 0.90 for all subjects). Of note, the young adults, on average, showed a CAR of around 0.96, a little less than what was observed on the older participants (i.e., CAR_OLD_ ∼0.98).

The CCI was in general low, for all participants, independently on their age. Muscle co-contraction was minimal when the subjects performed the MVIC test (CCI_HYA_ = 0.15 ± 0.21, CCI_OLD_ = 0.13 ± 0.10), and during the swing phase of the gait cycle (CCI_HYA_ <0.30 and CCI_OLD_<0.13). The maximum level of co-contraction was observed during the double support phase of the gait cycle (see Supplementary Material).

### 4.5 Gait assessment and biomechanical simulations

**FIGURE 3:**
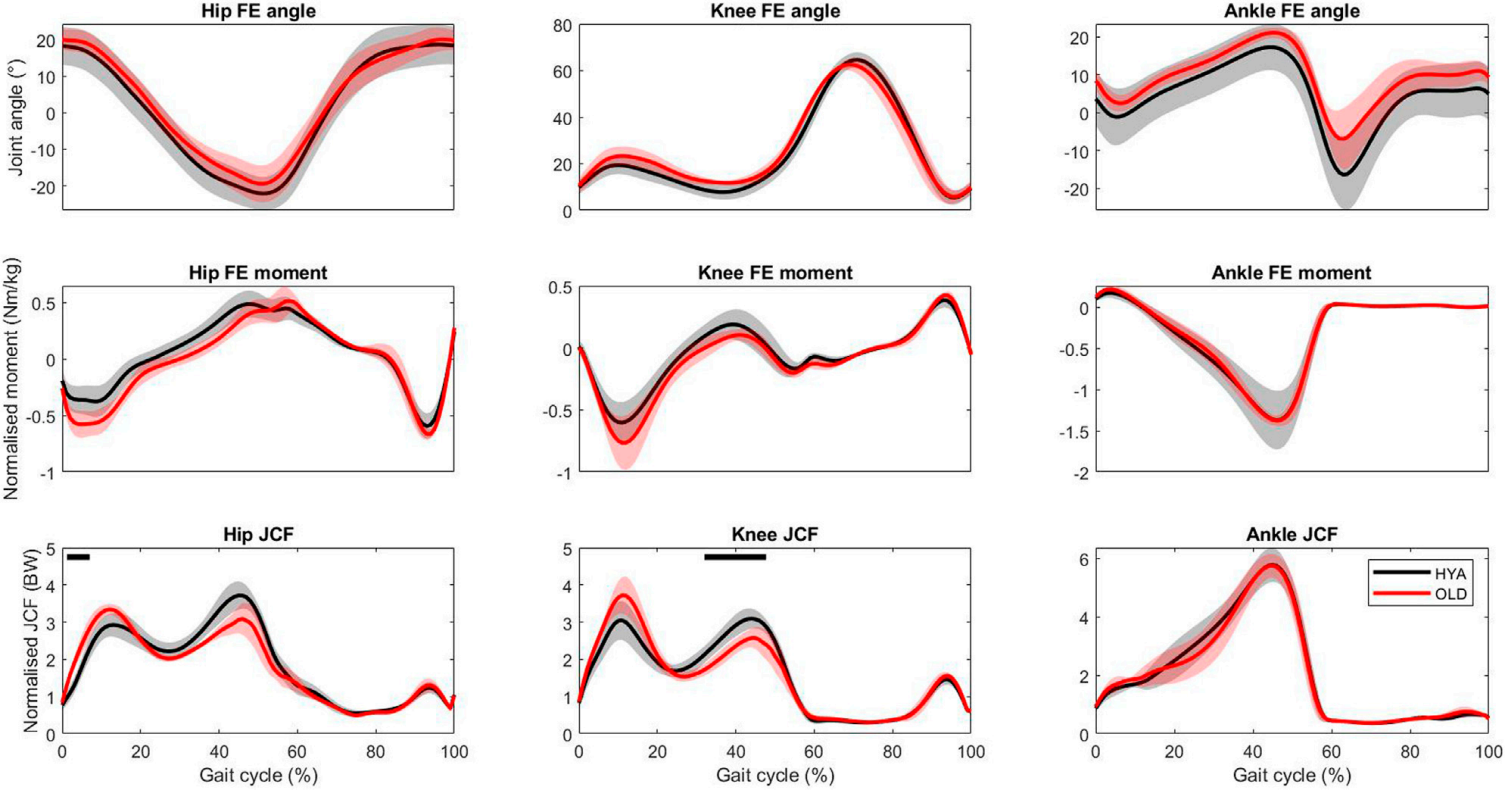
Hip, knee and ankle joint kinematics, kinetics and contact forces during overground walking predicted by the musculoskeletal models. The results are reported as mean (solid line) and standard deviation (shade) across each population (black = young adults, red = elderlies). Ten trials per participant were analysed. Joint contact force values are normalised to the body weight, to enable comparisons. The black bars on top identify statistical significance (*p* < 0.05) according to the SPM analysis.

All participants walked at a similar speed, with the elderly participants walking slightly faster than the young adults (1.32 ± 0.09 m/s vs. 1.25 ± 0.12 m/s, respectively), when instructed to select their preferred walking speed. Overall, young and older individuals exhibited similar joint kinematics, kinetics and contact force profiles (on the sagittal plane), as highlighted by the results of the simulations. However, statistically significant differences were found during the stance phase and the push-off phase of the gait cycle for what concerns the hip and knee joint contact forces, respectively (the models predicted larger second peaks for the HYA participants, compared to the elderlies. Figure 3).

## 5 Discussion

**FIGURE 4:**
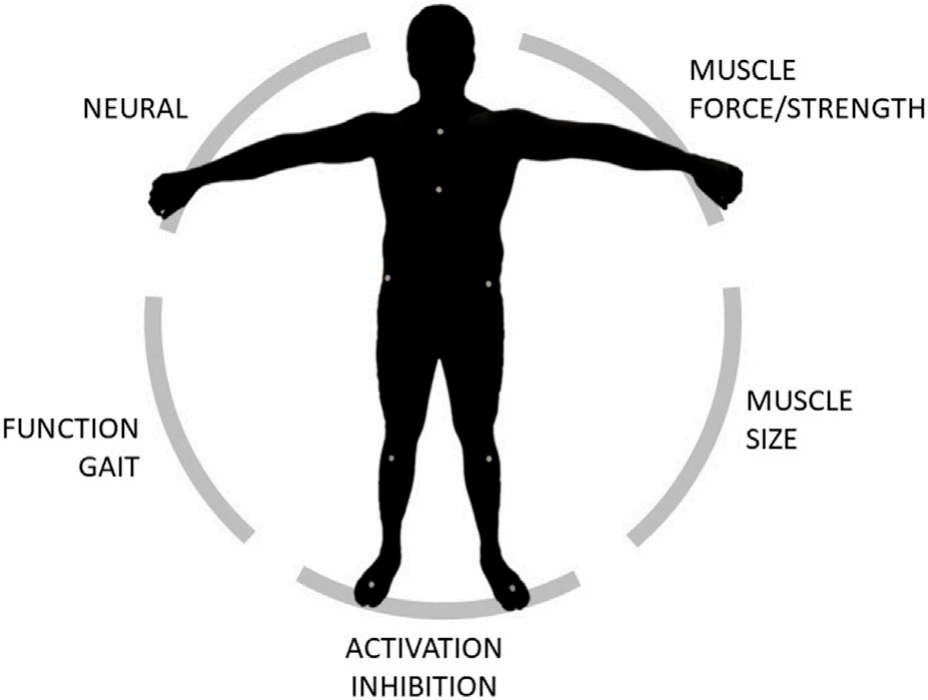
Schematic of the domains that can be extracted and/or explored through the proposed framework.

In this manuscript we proposed a framework that combines both experimental measurements and computational simulations to enable the full biomechanical characterization of an individual. Medical imaging, dynamometry, EMG and motion capture data were collected on 25 subjects (20 healthy young adults and 5 elderlies) to demonstrate the feasibility of the protocol. Furthermore, we indulged on the detailed description of the data elaboration and data analysis to enable replication and to highlight the type of information that can be extracted, allowing to get insights on five domains: loss of muscle mass (sarcopenia), loss of muscle force (dynapenia), presence of abnormal muscle activation patterns and muscle activation inhibition, loss of function (e.g., in terms of gait deviation) (Figure 4). All data were acquired and processed according to international guidelines.

The protocol took approximately 4 h and 30 min per subject, including the preparation time: 2 h30 for the dynamometry test, 1 h for the gait assessment and 30–60 min for the MRI acquisition. All participants completed the protocol and compliance to the protocol was high. Of note, the protocol can be easily tailored to the experimenter’s needs, and reduced in time e.g., performing the MVIC test in one single configuration (or focusing on one motion direction: leg extension or flexion), or complemented with additional tests (e.g., high-density EMG, ultrasound, isokinetic tests).

A comprehensive set of parameters was extracted, allowing to get insights on different biomechanical domains of clinical interest (Figure 4). The experimental findings are in line with previous studies. In particular, the maximal extension torques closely approximate those reported by O’Brien et al. (O’Brien et al., 2009), while the gait kinematics and kinetics is typical of a healthy adult population (Bovi et al., 2011; Moissenet et al., 2019; Rowe et al., 2021). In addition, as expected, and in accordance with previous studies (Rozand et al., 2020), none of the participants showed signs of poor muscle control or activation deficits (CCI<0.30 and CAR >0.96 in all cases, while performing the MVIC test at 75°), which could have been identified through the combination of electromyography and superimposed electrical stimulations. Indeed, the correct application of the stimulation is not trivial, as it is not uncommon to observe within- and between-subjects variability in terms of maximal torques profiles (despite analogous instructions and familiarization).

Furthermore, the predicted joint contact forces were within the normative ranges, i.e., approximately 3–4 N (van Rossom et al., 2018). Nonetheless, the use of musculoskeletal models enabled to appreciate some differences in both joint kinematics and joint loading during walking. This is quite important. Atypical contact force profiles and/or abnormal peak values are secondary to altered biomechanics and could serve as early predictor/indicator of neuromuscular pathologies (Pizzolato et al., 2017; Killen et al., 2020; Davico et al., 2022; Uhlrich et al., 2022).

Last, the availability of MRI data allowed for the reconstruction of 3D muscle volumes, better predictor of muscle strength than CSA measurements (Akagi et al., 2009). As highlighted in previous works (Trappe et al., 2001; Fuchs et al., 2023), elder participants had smaller muscles than their younger counterparts. With a larger dataset at hand or combining different datasets, population-specific atlases and robust regression models can be generated (e.g., Handsfield et al., 2014), which would expedite the process to estimate muscle volumes, thus, to identify and quantify the presence of sarcopenia. The possibility to detect fat infiltrations, which new algorithms promise to enable, would add more informative value. Alternatively, bioimpedentiometry measurements may be used to estimate the percentage of muscular and fat tissues.

Extending the investigation (and validation) of the proposed framework to other populations (e.g., subjects affected by musculoskeletal conditions and/or neuromuscular disorders) may lead in the long run to an improvement in the management of patients and consequently to an improvement in their general health and quality of life. From a clinical point of view, the discrimination of the determinants leading to muscle strength decline is essential to personalize clinical interventions. For example, if the tests show that the low muscle strength is mainly related to the loss of muscle mass nutritional and strength training may need to be implemented, while pharmacological interventions may be further required if the loss of muscle strength is mainly, or partly, related to abnormal or declined neural function.

To the authors’ knowledge, this is the first study showing such a comprehensive set of experimental measures and parameters, collected with the aim to fully characterize an individual from a biomechanical and neuromuscular standpoint. With a larger sample size at hand, one could apply data extraction and analytics approaches—eventually supported by machine learning or AI-based methods—to get insights into the mechanisms behind the loss of muscle force (Giarmatzis et al., 2020; Yeung et al., 2020; Liew et al., 2023; Moghadam et al., 2023; Rabbi et al., 2024).

The small sample size (and the limited number of elderly subjects enrolled) was the main limitation of this preliminary investigation, which was however devised to test the feasibility of the proposed workflow in a clinical setting.

There are some additional limitations to the study. For instance, as reported, the internal knee joint angle did not correspond to the theoretical angle of the dynamometer (see the reported internal angle *versus* nominal angle, Table 5). However, this is in line with previous works. The availability of a motion capture system would enable for a better control of the experiment, and/or to correct for movements. Moreover, the use of an isokinetic dynamometer to execute a MVIC test is the current gold standard. Second, the EMG data were—at times—noisy. This was due to the EMG system being wired, with the cables causing artifacts. However, the data elaboration pipeline allowed for a proper data cleaning. In addition, EMG data was also collected during a range of dynamic tasks, which enabled to compute important parameters, such as the CCI, during more common tasks/activities of daily living (including walking). Third, the groups were unbalanced and the numerosity was not sufficient to reach statistical significance for many comparisons (except for the results of the simulations), but this was not a primary objective for the study. A larger cohort and dataset would enable to use linear mixed models or decisional trees (e.g., classification and regression trees) to highlight any relationships/correlations between the extracted parameters. Fourth, the MSK models used in this study were generic models linearly scaled from a template model (Rajagopal et al., 2016; Uhlrich et al., 2022). Although their use was motivated by their application to two cohorts of healthy adults, and by an easier and faster development compared to personalized MSK models built off medical imaging data (Smith et al., 2015; Modenese et al., 2018; Princelle et al., 2023), the latter would yield more physiologically plausible estimate (particularly when employed to study individuals with MSK disorders) and may incorporate patient-specific characteristics (e.g., sarcopenia). Last, the criterion employed to estimate muscle forces and activations, which identified the solution that minimized the sum of squared muscle activations, may not be the most appropriate to simulate many motor tasks. However, as none of the participants was affected by/diagnosed with neuromuscular disorders, it was reasonable to assume that they performed a minimally demanding and easy task such as walking with minimal energy consumption. Also, it should be mentioned that the gait assessment was performed barefoot. Future studies should also investigate if some differences between barefoot or wearing shoes walking exist.

In conclusion, we hereby proposed a protocol and framework to enable the full biomechanical characterization of an individual, combining both *in vivo* and *in silico* approaches and we demonstrated its feasibility, in a clinical context. The same or similar framework can provide quantitative evidence to support clinical management and decision-making.

## Data Availability

The datasets presented in this article are not readily available because further processing and curation are required before any data can be publicly shared. The authors intend to share the curated dataset with the scientific community under the form of a data collection. Requests to access the datasets should be directed to giorgio.davico@unibo.it.
