# Supplementary material for: Towards a comprehensive biomechanical assessment of the elderly combining in vivo data and in silico methods
Source: Front Bioeng Biotechnol. 2024 May 6;12:1356417. doi: 10.3389/fbioe.2024.1356417 (PMC11102974; doi:10.3389/fbioe.2024.1356417)
Supplement: Supplementary file 1 [file Table1.docx]

Supplementary Material

Table S1 Individual maximal torques and force values from the dynamometry and hand-grip tests. The reported values correspond to the maximal values observed, across three trials.

|  | **MVIC torques [Nm]** | | | | **Hand-grip force**  **[kg]** | |
| --- | --- | --- | --- | --- | --- | --- |
| **ID** | **Extension** | | **Flexion** | |  |  |
|  | **75°** | **90°** | **75°** | **90°** |  |  |
| **HYA01** | 207.57 | 244.38 | 125.87 | 105.75 | 42.9 |  |
| **HYA02** | 159.60 | 184.95 | 68.49 | 61.10 | 29.2 |  |
| **HYA03** | 266.85 | 254.48 | 104.84 | 78.41 | 50 |  |
| **HYA04** | 226.79 | 207.96 | 93.75 | 77.24 | 50 |  |
| **HYA05** | 267.67 | 244.78 | 98.19 | 97.18 | 47 |  |
| **HYA06** | 344.58 | 344.54 | 120.73 | 103.27 | 50 |  |
| **HYA07** | 237.31 | 275.79 | 103.12 | 99.35 | 46 |  |
| **HYA08** | 215.26 | 176.93 | 79.26 | 67.95 | 28 |  |
| **HYA09** | 188.67 | 155.56 | 79.41 | 71.84 | 30 |  |
| **HYA10** | 251.87 | 222.99 | 99.81 | 89.17 | 40 |  |
| **HYA11** | 191.48 | 151.61 | 66.36 | 57.85 | 35 |  |
| **HYA12** | 128.08 | 121.00 | 72.09 | 64.75 | 30 |  |
| **HYA13** | 321.84 | 366.73 | 138.90 | 116.77 | 53 |  |
| **HYA14** | 220.02 | 234.25 | 96.87 | 85.44 | 33 |  |
| **HYA15** | 288.17 | 227.74 | 116.00 | 119.92 | 56 |  |
| **HYA16** | 163.67 | 150.26 | 70.72 | 57.20 | 36 |  |
| **HYA17** | 417.93 | 396.81 | 127.52 | 109.53 | 48 |  |
| **HYA18** | 237.70 | 182.20 | 96.58 | 86.85 | 35 |  |
| **HYA19** | 186.50 | 149.78 | 70.55 | 65.62 | 40 |  |
| **HYA20** | 199.55 | 164.34 | 69.29 | 54.46 | 32 |  |
| **OLD01** | 185.66 | 177.61 | 57.09 | 53.12 | 41 |  |
| **OLD02** | 245.84 | 211.84 | 94.69 | 80.80 | 47 |  |
| **OLD03** | 191.30 | 157.62 | 57.01 | 48.15 | 40.5 |  |
| **OLD04** | 170.73 | 152.59 | 64.60 | 53.74 | 35 |  |
| **OLD05** | 122.19 | 103.11 | 48.67 | 47.36 | 20.5 |  |

Table S Peak knee joint contact forces predicted by the musculoskeletal models during the overground walking trials. The reported values, in BW, correspond to the mean peak(s) across the ten analyzed repetitions/simulations.

|  | **Predicted knee joint contact forces** | |  |
| --- | --- | --- | --- |
| **ID** | **1^st^ peak [BW]** | **2^nd^ peak [BW]** | |
| **HYA01** | 3.20 | 3.17 | |
| **HYA02** | 3.56 | 3.11 | |
| **HYA03** | 3.10 | 2.71 | |
| **HYA04** | 2.46 | 3.20 | |
| **HYA05** | 3.35 | 3.17 | |
| **HYA06** | 3.32 | 2.86 | |
| **HYA07** | 2.98 | 2.98 | |
| **HYA08** | 2.74 | 2.70 | |
| **HYA09** | 2.70 | 3.64 | |
| **HYA10** | 3.47 | 3.17 | |
| **HYA11** | 3.22 | 3.04 | |
| **HYA12** | 4.51 | 3.28 | |
| **HYA13** | 3.58 | 3.11 | |
| **HYA14** | 2.15 | 3.26 | |
| **HYA15** | 3.21 | 3.20 | |
| **HYA16** | 2.98 | 3.30 | |
| **HYA17** | 3.02 | 3.60 | |
| **HYA18** | 2.56 | 3.14 | |
| **HYA19** | 2.46 | 3.21 | |
| **HYA20** | 3.04 | 2.90 | |
| **OLD01** | 4.55 | 2.48 | |
| **OLD02** | 3.76 | 3.14 | |
| **OLD03** | 3.95 | 2.43 | |
| **OLD04** | 3.29 | 2.46 | |
| **OLD05** | 3.11 | 2.61 | |

Table S3 Co-contraction index computed for each subject from the surface EMG data collected during the overground walking trials in the gait laboratory, split per phases of the gait cycle. The results are reported as mean across the 10 available trials per subject. For details on the processing methods, please refer to the main text.

| **ID** | **Initial  double support**  **(0-10%)** | **Single  support**  **(10-50%)** | **Pre-swing phase**  **(50-60%)** | **Swing**  **phase**  **(60-100%)** |
| --- | --- | --- | --- | --- |
| **HYA01** | 0.19 | 0.05 | 0.08 | 0.17 |
| **HYA02** | 0.03 | 0.10 | 0.07 | 0.04 |
| **HYA03** | 0.06 | 0.08 | 0.19 | 0.08 |
| **HYA04** | 0.11 | 0.06 | 0.05 | 0.09 |
| **HYA05** | 0.40 | 0.11 | 0.07 | 0.30 |
| **HYA06** | 0.55 | 0.13 | 0.03 | 0.12 |
| **HYA07** | 0.12 | 0.16 | 0.04 | 0.05 |
| **HYA08** | 0.03 | 0.07 | 0.03 | 0.05 |
| **HYA09** | 0.07 | 0.06 | 0.07 | 0.19 |
| **HYA10** | 0.07 | 0.05 | 0.06 | 0.12 |
| **HYA11** | 0.04 | 0.06 | 0.11 | 0.09 |
| **HYA12** | 0.16 | 0.12 | 0.21 | 0.24 |
| **HYA13** | 0.07 | 0.11 | 0.31 | 0.16 |
| **HYA14** | 0.08 | 0.17 | 0.08 | 0.10 |
| **HYA15** | 0.08 | 0.24 | 0.45 | 0.20 |
| **HYA16** | 0.10 | 0.22 | 0.12 | 0.07 |
| **HYA17** | 0.12 | 0.18 | 0.10 | 0.06 |
| **HYA18** | 0.12 | 0.12 | 0.22 | 0.11 |
| **HYA19** | 0.10 | 0.20 | 0.25 | 0.15 |
| **HYA20** | 0.21 | 0.25 | 0.19 | 0.22 |
| **OLD01** | 0.08 | 0.27 | 0.21 | 0.08 |
| **OLD02** | 0.05 | 0.32 | 0.30 | 0.06 |
| **OLD03** | 0.18 | 0.29 | 0.26 | 0.11 |
| **OLD04** | 0.16 | 0.41 | 0.33 | 0.13 |
| **OLD05** | 0.26 | 0.27 | 0.06 | 0.10 |
